# ASA class is independently associated with 30-day mortality after mild traumatic brain injury with intracranial injury: a national retrospective cohort study

**DOI:** 10.1007/s00701-026-06900-9

**Published:** 2026-05-09

**Authors:** Olivia Kiwanuka, Hans Pettersson, Gabriel Sandblom

**Affiliations:** 1 Department of Clinical Science and Education, Karolinska Institutet, Södersjukhuset, Stockholm, Sweden; 2 Department of Surgery, Stockholm South General Hospital, Stockholm, Sweden

**Keywords:** Mild traumatic brain injury, Trauma, ASA class, Comorbidity, Mortality, Prognostic validation

## Abstract

**Introduction:**

Outcome assessment after mild traumatic brain injury (mTBI) has largely focused on acute injury severity, while the role of pre-injury health has received comparatively less attention. The American Society of Anesthesiologists (ASA) physical status classification provides a simple measure of pre-existing health and comorbidity that may be relevant to early outcomes after trauma. This study examined whether pre-injury ASA class is independently associated with short-term mortality after mTBI and compared these associations with those observed in trauma patients without brain injury (NTBI).

**Methods:**

We conducted a nationwide retrospective study using the Swedish Trauma Registry (2018–2023). Adults (≥ 18 years) with Glasgow Coma Scale (GCS) > 12 were included and classified as mTBI (ICD-10 S06.0–S06.9) or NTBI (no S06 diagnosis, AIS head = 0, age-matched). The primary outcome was 30-day all-cause mortality. Associations between clinical variables and mortality were examined using univariable and multivariable logistic regression, with models specified a priori to separate age–ASA effects from injury-related adjustment.

**Results:**

A total of 8,670 patients were included in the mTBI cohort and 26,001 in the age-matched cohort. The mTBI cohort had poorer health (ASA ≥ 3: 27% vs 24%), and more often injured by low-energy falls (28% vs 20%). Mortality was higher in mTBI (5% vs 2.8%), and outcomes poorer (Glasgow Outcome Scale 1–3: 23% vs 17%).

Within the mTBI cohort, mortality was strongly associated with increasing age, higher ASA category, and greater intracranial injury severity. After adjustment for age and injury-related variables, ASA class remained independently associated with 30-day mortality. Specifically, compared to ASA 1 patients, those with ASA 2 had 2.8 times higher odds of 30-day mortality, ASA 3 patients had 4.3 times higher odds, and ASA 4 patients had 21 times higher odds. A graded association between ASA class and mortality was also observed in the NTBI cohort, although the pattern of injury-related covariates differed.

**Conclusions:**

Pre-injury health status, measured by the ASA class, was independently associated with 30-day mortality in patients with mild traumatic brain injury. Comparable associations in non-TBI trauma suggest that comorbidity and baseline health contribute substantially to early post-trauma mortality among awake trauma patients. ASA class may provide complementary information to traditional injury-based assessments, particularly in older patients, although further studies are needed to evaluate how such information can best inform clinical decision-making.

**Supplementary Information:**

The online version contains supplementary material available at 10.1007/s00701-026-06900-9.

## Introduction

Mild traumatic brain injury (mTBI), defined by a Glasgow Coma Scale (GCS) of 13–15, accounts for the majority of traumatic brain injuries worldwide, representing approximately 50–95% of the estimated 55 million cases each year [13]. Once considered an injury of the young, TBI is now increasingly seen among older and frail adults. This demographic shift has been accompanied by higher mortality, longer recovery time, and greater demand for healthcare resources [24, 28].

Historically low mortality rates in mTBI have led most outcome research and prognostic frameworks in traumatic brain injury to focus on moderate-to-severe injuries. These frameworks largely emphasize acute physiological derangement and radiological injury severity, while pre-injury health status and comorbidity have received comparatively less attention [17, 23]. With the increase of the elderly trauma population understanding how pre-existing health influences outcome even after seemingly “mild” injuries has become increasingly important.

We have previously shown that pre-injury health and anesthesia risk assessed with the American Society of Anesthesiologists (ASA) physical status classification system is associated with poorer long-term health-related quality of life after mTBI [7], and with mortality across all TBI severities [8, 9]. Building on this work, the present study examines whether ASA class is independently associated with short-term mortality after mTBI in a large, nationwide cohort.

To place these findings in context, we also examined a comparison cohort of trauma patients without brain injury (NTBI). This comparison allows assessment of whether the observed association between pre-injury health and mortality is specific to intracranial injury or reflects a broader vulnerability among comorbid trauma patients. By contrasting these groups, we aim to clarify the impact of pre-injury health to early mortality and to inform discussions on how comorbidity may be considered alongside traditional injury severity measures in contemporary trauma care.

## Method

### Study design

This was a retrospective, nationwide cohort study based on data from the Swedish Trauma Registry (SweTrau), covering the period January 1, 2018, to December 31, 2023. The study was approved by the Swedish Ethical Review Authority (Dnr: 2025—02316—01). The primary endpoint was 30-day all-cause mortality following trauma.

### Study population and variable definitions

All adult patients (≥ 18 years) registered in SweTrau during the study window were screened. SweTrau is a national quality registry established in 2011 by the Swedish Trauma Society, based on the revised Utstein template. Inclusion criteria are trauma team activation, NISS ≥ 15, or transfer to a participating hospital within 7 days. Exclusions include trauma alerts without trauma and isolated chronic subdural hematoma. Demographic and injury data were obtained directly from the registry. GCS was assessed by the attending clinician at admission. Abbreviated Injury Scale (AIS) coding followed the 2005 Update 2008 version and was performed by certified registrars. An AIS head score of 0 indicates no head injury, while AIS head score 1 reflects minor or superficial head injuries without intracranial involvement. ASA class represents pre-injury health status as assessed from medical records. Polytrauma was defined as AIS > 2 in two or more body regions, isolated TBI was defined as head injury with all other regions AIS ≤ 1.

For individuals with multiple trauma events, only the first registration per patient was included. Patients with missing trauma date or missing ASA classification were excluded.

To focus on conscious trauma patients, both cohorts required GCS 13—15 on hospital admission. Patients were then divided into two mutually exclusive groups:

1. mTBI cohort: ICD-10 diagnosis S06.0–S06.9, regardless of other injuries (Supplementary Table 1).
2. NTBI cohort: No S06 diagnosis and AIS head = 0.

Patients not meeting criteria for either group were excluded from comparative analyses.

To minimize confounding by age, the NTBI cohort was age-matched to the mTBI group. Matching was performed using exact frequency matching by integer years of age. For each age represented in the mTBI cohort, up to three NTBI patients of the same age were randomly selected. When fewer than three NTBI patients were available for a given age, all available cases were included. This ensured an identical age distribution between the mTBI and matched NTBI cohorts while preserving the overall characteristics of the NTBI population.

### Statistical analysis

Descriptive statistics and unadjusted comparisons presented in Tables 1, 2 and 3 were used to characterize the study cohorts and to illustrate crude differences in case mix and outcomes across clinically relevant categories. Where reported, p-values reflect unadjusted comparisons and are intended to support descriptive interpretation rather than formal hypothesis testing or variable selection. Multivariable analyses were specified a priori to estimate conditional associations between pre-injury ASA class and mortality, and interpretation focuses on the magnitude and consistency of adjusted effect estimates rather than statistical significance alone.

**Table 1:** Baseline demographic and injury characteristics of patients with mild traumatic brain injury (mTBI) and non–traumatic brain injury (NTBI)

	mTBI *N* = 8,670^a^	NTBI *N* = 26,001^a^	*p*-value^b^
Age	58 [38, 75]	58 [38, 75]	> 0.9
Gender (male)	5,496 (63%)	16,692 (64%)	0.2
ASA class			< 0.001
ASA 1	3,660 (42%)	11,680 (45%)	
ASA 2	2,635 (30%)	7,856 (30%)	
ASA 3	2,255 (26%)	6,103 (23%)	
ASA 4 +	120 (1.4%)	362 (1.4%)	
Mechanism of injury			< 0.001
Traffic	3,234 (37%)	11,218 (43%)	
Falls, high energy	2,192 (25%)	5,394 (21%)	
Falls, low energy	2,434 (28%)	5,127 (20%)	
Penetrating	80 (0.9%)	2,256 (8.7%)	
Other	730 (8.4%)	2,006 (7.7%)	
Transport to hospital			< 0.001
Walk-in	230 (2.7%)	709 (2.7%)	
Private/public vehicle	207 (2.4%)	692 (2.7%)	
Ground ambulance	7,255 (84%)	22,014 (85%)	
Helicopter ambulance	390 (4.5%)	1,088 (4.2%)	
Other	114 (1.3%)	357 (1.4%)	
Unknown	474 (5.5%)	1,141 (4.4%)	
GCS			< 0.001
13	722 (8.3%)	610 (2.3%)	
14	1,977 (23%)	2,084 (8.0%)	
15	5,971 (69%)	23,307 (90%)	
Hypotension	135 (1.6%)	791 (3.0%)	< 0.001
Unknown	142 (1.6%)	416 (1.6%)	
NISS	12 [5, 22]	5 [2, 12]	< 0.001
Unknown	0	178	
AIS head			< 0.001
0	2 (< 0.1%)	26,001 (100%)	
1	2,061 (24%)	0 (0%)	
2	2,811 (32%)	0 (0%)	
3	2,556 (29%)	0 (0%)	
4	732 (8.4%)	0 (0%)	
5	508 (5.9%)	0 (0%)	
GOS at discharge			< 0.001
Good recovery	3,280 (38%)	12,806 (49%)	
Moderate disability	3,392 (39%)	8,450 (32%)	
Severe disability	1,650 (19%)	4,008 (15%)	
Vegetative state	17 (0.2%)	23 (< 0.1%)	
Death	295 (3.4%)	503 (1.9%)	
Unknown	36 (0.4%)	211 (0.8%)	
30-day mortality	433 (5.0%)	735 (2.8%)	< 0.001

Comparison of demographic and injury characteristics between patients with mild traumatic brain injury (mTBI) and those without TBI (NTBI). Patients with mTBI were older, had poorer pre-injury health, and higher mortality than the NTBI group. Values are presented as median [IQR] or n (%). *ASA* American Society of Anesthesiologists physical status classification, *GCS* Glasgow Coma Scale, *NISS *New Injury Severity Score, *GOS* Glasgow Outcome Scale, *LEF* low-energy fall, *HEF* high-energy fall, *TBI* traumatic brain injury

^a^*n* (%)

^b^Wilcoxon rank sum test; Pearson's Chi-squared test

**Table 2:** Hospital course and discharge characteristics of patients with mild traumatic brain injury (mTBI) and non–traumatic brain injury (NTBI)

	mTBI *N* = 8,670^a^	NTBI *N* = 26,001^a^	*p*-value^b^
Time to hospital (min)	71 [49, 135]	77 [53, 158]	** < 0.001**
Transport to hospital			** < 0.001**
Walk-in	230 (2.7%)	709 (2.7%)	
Private/public vehicle	207 (2.4%)	692 (2.7%)	
Ground ambulance	7,255 (84%)	22,014 (85%)	
Helicopter ambulance	390 (4.5%)	1,088 (4.2%)	
Other	114 (1.3%)	357 (1.4%)	
Trauma team activated			** < 0.001**
Full	1,931 (22%)	6,938 (27%)	
Limited	1,882 (22%)	2,872 (11%)	
No	4,849 (56%)	16,160 (62%)	
Time to CT (min)	50 [31, 98]	51 [33, 90]	0.8
Not performed	249	2,857	
Hospital care level			
ED	1,899 (22%)	9,510 (37%)	
General Ward	3,794 (44%)	7,699 (30%)	
HDU	1,071 (12%)	2,492 (9.6%)	
ICU	1,580 (18%)	3,116 (12%)	
Hospital stay (days)	3 [2, 6]	2 [1, 6]	** < 0.001**
Discharge destination			** < 0.001**
Home	6,286 (73%)	19,362 (74%)	
Rehabilitation	1,136 (13%)	2,818 (11%)	
Other intermediate ward	531 (6.1%)	1,722 (6.6%)	
Other ICU	228 (2.6%)	440 (1.7%)	
Other	189 (2.2%)	1,032 (4.0%)	
Morgue	292 (3.4%)	502 (1.9%)	
Transferred status			** < 0.001**
None	7,553 (87%)	22,809 (88%)	
Transferred both in and out	415 (4.8%)	839 (3.2%)	
Transferred IN	169 (1.9%)	490 (1.9%)	
Transferred OUT	529 (6.1%)	1,755 (6.7%)	

Hospital course and discharge characteristics of patients with mTBI and NTBI. Compared with NTBI, mTBI patients had longer hospital stays, more intensive care admissions, and higher mortality. Values are presented as median [IQR] or n (%). *ASA* American Society of Anesthesiologists, *CT* computed tomography, *HDU* high-dependency unit, *ICU* intensive care unit, *CCU* coronary care unit, *TBI* traumatic brain injury

^a^*n* (%) ^b^Wilcoxon rank sum test; Pearson's Chi-squared test

**Table 3:** Baseline characteristics and hospital outcomes of survivors and non-survivors within the mild traumatic brain injury (mTBI) cohort

	Alive *N* = 8,237^a^	Deceased *N* = 433^a^	*p*-value^b^
Age	57 [37, 73]	83 [77, 89]	** < 0.001**
Gender (male)	5,227 (63%)	269 (62%)	0.6
ASA class			** < 0.001**
ASA 1	3,646 (44%)	14 (3.2%)	
ASA 2	2,534 (31%)	101 (23%)	
ASA 3	1,980 (24%)	275 (64%)	
ASA 4 +	77 (0.9%)	43 (9.9%)	
MOI			** < 0.001**
Traffic	3,190 (39%)	44 (10%)	
Falls, high energy	2,098 (25%)	94 (22%)	
Falls, low energy	2,155 (26%)	279 (64%)	
Penetrating	80 (1.0%)	0 (0%)	
Other	714 (8.7%)	16 (3.7%)	
GCS			** < 0.001**
13	629 (7.6%)	93 (21%)	
14	1,845 (22%)	132 (30%)	
15	5,763 (70%)	208 (48%)	
Hypotension	77 (1.0%)	7 (1.7%)	**0.2**
AIS head			** < 0.001**
0—1	2,047 (25%)	16 (3.7%)	
2	2,741 (33%)	70 (16%)	
3	2,414 (29%)	142 (33%)	
4	658 (8.0%)	74 (17%)	
5	377 (4.6%)	131 (30%)	
NISS	12 [5, 21]	25 [17, 33]	** < 0.001**
Isolated TBI	3,258 (40%)	220 (51%)	** < 0.001**
EDH	318 (3.9%)	17 (3.9%)	** > 0.9**
SDH	2,349 (29%)	266 (61%)	** < 0.001**
trSAH	2,357 (29%)	220 (51%)	** < 0.001**
ICH	260 (3.2%)	50 (12%)	** < 0.001**
Contusion	1,270 (15%)	89 (21%)	**0.005**
Polytrauma	120 (1.5%)	16 (3.7%)	** < 0.001**
Hospital care level			** < 0.001**
ED	1,876 (24%)	23 (5.6%)	
General Ward	3,602 (45%)	192 (46%)	
HDU	1,027 (13%)	44 (11%)	
ICU	1,426 (18%)	154 (37%)	
Unknown	306	20	
Hospital stay (days)	3.0 [2.0, 6.0]	5.0 [3.0, 10.0]	** < 0.001**

Comparison of baseline characteristics and hospital outcomes between survivors and non-survivors within the mTBI cohort. Non-survivors were older, had higher ASA class, lower GCS, and more severe intracranial injuries. Values are presented as median [IQR] or n (%)

*ASA* American Society of Anesthesiologists, *GCS* Glasgow Coma Scale, *NISS* New Injury Severity Score, *AIS* Abbreviated Injury Scale, *LEF* low-energy fall, I*CU* intensive care unit, *GOS* Glasgow Outcome Scale, *TBI* traumatic brain injury, *EDH* epidural hematoma, *SDH* subdural hematoma, *trSAH* traumatic subarachnoid hemorrhage, *ICH* intracerebral hemorrhage

^a^*n* (%) ^b^Wilcoxon rank sum test; Pearson's Chi-squared test

Associations between clinical characteristics and 30-day mortality in patients with mTBI were examined using logistic regression, with 30-day all-cause mortality modelled as a binary outcome. Age was analyzed as a continuous variable. Pre-injury health status was categorized using ASA class. GCS was analyzed as a categorical variable. Head injury severity was assessed using the AIS head; AIS scores of 0 and 1 were combined into a single reference category due to sparse observations in AIS 0. Low-energy fall, isolated traumatic brain injury, and polytrauma were analyzed as binary variables. Crude associations were estimated using separate unadjusted logistic regression models for each variable, and odds ratios (ORs) with 95% confidence intervals (CIs) were calculated. ASA class, GCS, and AIS head were modeled as categorical variables to avoid imposing a linear effect across ordered levels, as preliminary analyses indicated non-linear increases in mortality across categories. Sensitivity analyses using ordinal trend coding yielded similar overall results.

To evaluate the robustness of associations after adjustment, two multivariable logistic regression models were specified a priori. Model 1 included age and ASA class. Model 2 (full model) included age, ASA class, GCS category, AIS head category, low-energy fall, isolated traumatic brain injury, and polytrauma. Multivariable analyses were conducted using complete-case analysis. Descriptive data presented alongside adjusted estimates (number of deaths, total number of observations, and within-level mortality) were derived from the same complete-case dataset used for the full multivariable model to ensure consistency between descriptive and adjusted results. Odds ratios with 95% confidence intervals are reported, and reference categories are explicitly indicated in the tables.

For the NTBI cohort, AIS head was zero by definition and therefore not included. Instead, overall injury severity was represented by the New Injury Severity Score (NISS). The same modelling strategy was applied. Analyses were restricted to complete cases. No model selection procedures were applied, and all variables were chosen based on prior clinical relevance and previous literature. All statistical analyses were performed using R version 4.5.0 (R Foundation for Statistical Computing, Vienna, Austria) within the RStudio environment (RStudio 2025.09.2, Posit Software, PBC).

## Results

### Study population

**Fig. 1:**
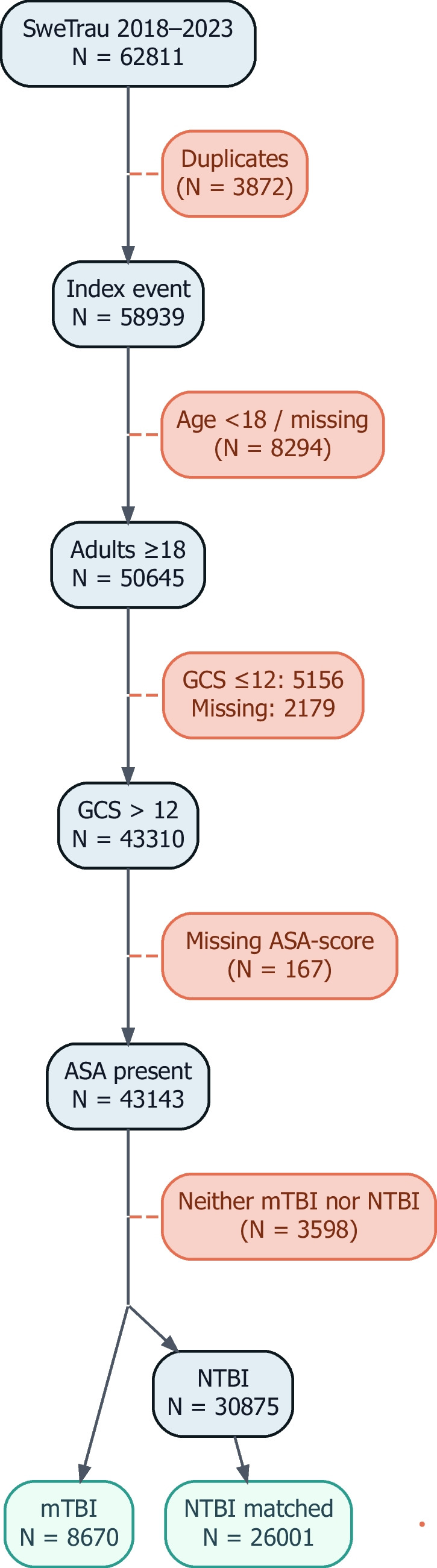
Flow chart of patient selection and cohort formation. Study flowchart showing inclusion and exclusion of patients with mTBI from the Swedish National Trauma Registry (2018–2023). Abbreviations: ASA = American Society of Anesthesiologists physical status classification; GCS = Glasgow Coma Scale

During the study period, 8, 670 patients with mTBI met the inclusion criteria, and 26, 001 patients in the age matched NTBI control group (Fig. 1).

### Demographic characteristics

The median age was 58 and a majority of the patients were male in both groups, (63% vs 64%, *p* = 0.2). The mTBI cohort had poorer pre-injury health, with ASA ≥ 3 in 27% vs 25% (*p* < 0.001) (Table 1).

Mechanisms of injury differed between the cohorts. Low-energy falls (LEF) accounted for 28% of mTBI but only 20% of NTBI, while traffic-related mechanisms dominated in NTBI (43% vs 37%, *p* < 0.001). Penetrating trauma (stabbing, gunshot) was rare in mTBI (0.9%) but more common in NTBI (8.7%).

The mTBI cohort presented more often with GCS 13–14 (31% vs 10%) and had higher NISS (median 12 vs 5, *p* < 0.001). Complicated mTBI was seen in 76% of the mTBI cohort, with 5.9% having an AIS head of 5. By definition 100% of NTBI cohort had an AIS head of 0. GOS at discharge was poorer, (23% vs 17% GOS 1—3) and mortality rate was higher in the mTBI cohort (5% vs 2.8%).

Hospital pathways also differed between groups (Table 2). The mTBI cohort had slightly lower time to hospital (71min vs 77 min, *p* < 0.001), fewer trauma team activations (22% vs 27, *p* < 0.001), but similar times to radiology (50min vs 51 min, *p* = 0.8). The mTBI patients were more often treated at higher-level units, with 18% admitted to ICU versus 12% for NTB, had slightly longer hospital stay, and fewer were discharged home.

### Mortality and injury characteristics within mTBI

**Fig. 2:**
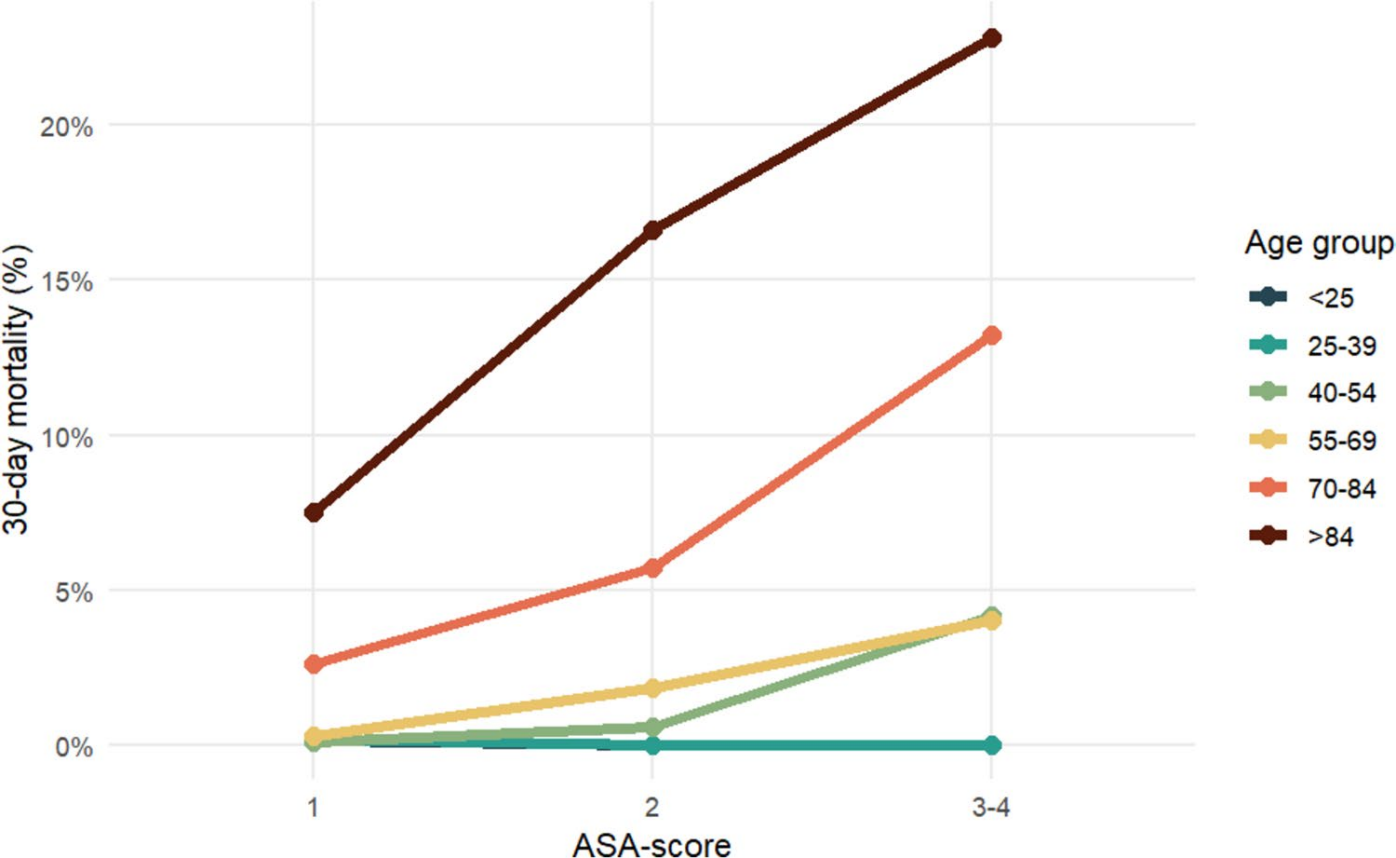
Crude 30-day mortality by ASA score and age group in patients with mild traumatic brain injury. Thirty-day mortality after mTBI stratified by ASA score and age group. Mortality increased stepwise with higher ASA categories across all age groups, with the steepest rise observed among the oldest patients (> 84 years). Abbreviations: ASA = American Society of Anesthesiologists physical status classification

Among patients with mTBI, 433 (5%) died within 30 days (Table 3, Fig. 2). Non-survivors were substantially older (median 83 vs 57 years, *p* < 0.001) and had poorer pre-injury health (ASA ≥ 3 in 74% vs 26%, *p* < 0.001).

The main mechanisms of injury differed between the groups with LEF dominating among the deceased (66% vs 26%), and traffic injuries in the surviving cohort (39% vs 11%). Non-survivors had lower GCS (13–14 in 51% vs 29%) and higher AIS head scores (AIS ≥ 4 in 47% vs 13%). Severe intracranial lesions such as subdural (SDH) (61% vs 29%) and subarachnoid (trSAH) hemorrhage (51% vs 29%), were markedly more common among those who died. ICU admission was frequent (37% vs 18%), and median hospital stay was longer (5 vs 3 days, *p* < 0.001).

### Associations with 30-day mortality

In patients with mTBI, several clinical variables were associated with 30-day mortality in crude analyses (Table 4). Increasing age and poorer pre-injury health, as measured by higher ASA category, showed strong graded associations with mortality. Mortality increased from 0.4% in ASA class 1 to 35.8% in ASA class ≥ 4, corresponding to a crude odds ratio (OR) of 145.0 (95% CI 78.0–285.2) for ASA ≥ 4 compared with ASA 1. Lower GCS, increasing intracranial injury severity (AIS head), polytrauma, isolated TBI, and low-energy fall mechanism appeared associated with 30-day mortality in crude analyses.

**Table 4:** Associations with 30-day mortality in patients with mild traumatic brain injury (mTBI)

Variables	Level	Deaths/N	Mortality %	Crude OR (95% CI)	Adjusted OR (95% CI) Model 1: Age + ASA	Adjusted OR (95% CI) Model 2: All variables
ASA class	1	14/3649	0.4%	1.00 (ref)	1.00 (ref)	1.00 (ref)
	2	100/2619	3.8%	10.31 (6.09–18.88)	3.20 (1.86–5.94)	2.82 (1.61–5.31)
	3	270/2238	12.1%	35.62 (21.59–64.10)	5.55 (3.25–10.24)	4.35 (2.51–8.13)
	4 +	43/120	35.8%	145.00 (78.01–285.23)	25.27 (13.15–51.08)	21.19 (10.69–44.03)
GCS	15	207/5958	3.5%	1.00 (ref)	-	1.00 (ref)
	14	129/1959	6.6%	1.96 (1.56–2.45)	-	1.42 (1.10–1.83)
	13	91/709	12.8%	4.09 (3.14–5.29)	-	3.08 (2.26–4.17)
AIS head	0—1	16/2061	0.8%	1.00 (ref)	-	1.00 (ref)
	2	68/2801	2.4%	3.18 (1.89–5.69)	-	2.50 (1.43–4.60)
	3	139/2537	5.5%	7.41 (4.54–12.96)	-	2.76 (1.60–5.04)
	4	73/725	10.1%	14.31 (8.50–25.62)	-	4.33 (2.43–8.13)
	5	131/502	26.1%	45.13 (27.37–79.65)	-	15.88 (8.92–29.79)
Low-energy fall	No	148/6192	2.4%	1.00 (ref)	-	1.00 (ref)
	Yes	279/2434	11.5%	5.29 (4.31–6.51)	-	1.18 (0.91–1.53)
Isolated TBI	No	210/5176	4.1%	Ref	-	1.00 (ref)
	Yes	217/3450	6.3%	1.59 (1.31–1.93)	-	0.89 (0.67–1.18)
Polytrauma	No	337/7786	4.3%	1.00 (ref)	-	1.00 (ref)
	Yes	90/840	10.7%	2.65 (2.07–3.37)	-	2.23 (1.58–3.17)

Crude mortality, crude odds ratios (ORs), and adjusted ORs with 95% confidence intervals (CIs) are shown. Model 1 includes age and ASA class. Model 2 (full model) includes age, ASA class, Glasgow Coma Scale (GCS), AIS head score (0–1 as reference), low-energy fall, isolated TBI, and polytrauma. Deaths/N and mortality percentages are calculated from the complete-case dataset used for the full model. Reference categories are indicated as 1.00 (ref)

*ASA* American Society of Anesthesiologists, *GCS* Glasgow Coma Scale, *AIS* Abbreviated Injury Scale, *TBI* traumatic brain injury, *OR* odds ratio, *CI* confidence interval

After adjustment for age and ASA (Model 1), the magnitude of the association between ASA and mortality was substantially attenuated but remained statistically significant across all ASA categories. Further adjustment for injury-related variables in the fully adjusted model (Model 2) resulted in only modest additional attenuation, with ASA class ≥ 4 remaining strongly associated with mortality (adjusted OR 21.2, 95% CI 10.7–44.0). In the fully adjusted model, lower GCS, increasing AIS head category, and polytrauma remained independently associated with higher mortality. Low-energy fall mechanism and isolated TBI were no longer independently associated with mortality after full adjustment.

In the NTBI cohort, a similar pattern was observed in crude analyses (Table 5). Increasing age and higher ASA category were strongly associated with mortality, with mortality rising from 0.2% in ASA class 1 to 25.9% in ASA class ≥ 4 (crude OR 209.5, 95% CI 134.2–335.0). Lower GCS, low-energy fall mechanism, and polytrauma were also associated with increased crude mortality. In contrast to the mTBI cohort, the crude association between polytrauma and mortality was weaker, and isolated TBI was not applicable.

**Table 5:** Associations with 30-day mortality in non–traumatic brain injury (NTBI) trauma patients

Variables	Level	Deaths/N	Mortality %	Crude OR (95% CI)	Adjusted OR (95% CI) Model 1: ASA + Age	Adjusted OR (95% CI) Model 2: All variables
ASA class	1	34/11,540	0.3%	1.00 (ref)	1.00 (ref)	1.00 (ref)
	2	166/7807	2.1%	7.35 (5.15–10.82)	1.93 (1.35–2.84)	2.02 (1.39–3.01)
	3	428/6044	7.1%	25.79 (18.46–37.30)	3.55 (2.51–5.17)	3.43 (2.39–5.08)
	4 +	99/361	27.4%	127.87 (85.88–194.84)	18.12 (12.03–27.81)	19.37 (12.61–30.35)
GCS	15	512/23,107	2.2%	1.00 (ref)	-	1.00 (ref)
	14	141/2049	6.9%	3.26 (2.68–3.94)	-	1.70 (1.37–2.10)
	13	74/596	12.4%	6.26 (4.80–8.05)	-	4.24 (3.14–5.68)
Low-energy fall	No	303/20,670	1.5%	1.00 (ref)	-	1.00 (ref)
	Yes	424/5082	8.3%	6.12 (5.26–7.12)	-	1.21 (1.01–1.46)
Polytrauma	No	672/24,868	2.7%	1.00 (ref)	-	1.00 (ref)
	Yes	55/884	6.2%	2.39 (1.78–3.14)	-	0.89 (0.62–1.26)

Crude mortality, crude odds ratios (ORs), and adjusted ORs with 95% confidence intervals (CIs) are shown. Model 1 includes age and ASA class. Model 2 (full model) includes age, ASA class, GCS, low-energy fall, New Injury Severity Score (NISS), and polytrauma. Deaths/N and mortality percentages are calculated from the complete-case dataset used for model 2. Reference categories are indicated as 1.00 (ref)

*ASA* American Society of Anesthesiologists, *GCS* Glasgow Coma Scale, *NISS* New Injury Severity Score, *OR* odds ratio, *CI* confidence interval

After adjustment for age and ASA (Model 1), the associations between ASA category and mortality were markedly attenuated but remained statistically significant. In the fully adjusted model (Model 2), ASA category, lower GCS, and low-energy fall mechanism remained independently associated with mortality, whereas polytrauma was no longer independently associated with outcome.

## Discussion

In this nationwide cohort of patients with mTBI, pre-injury health status, as assessed by the ASA class, was independently associated with 30-day mortality after adjustment for age, with only modest additional attenuation after further adjustment for injury-related variables. This confirms and extends our earlier single-center findings, where ASA ≥ 3 was likewise linked to increased 90-day mortality after complicated mTBI [9]. By including both mTBI and an age-matched NTBI trauma population, the present study confirms an independent association between ASA class and 30-day mortality in a larger, more heterogeneous cohort.

Although mTBI is often viewed as a relatively minor injury, the observed mortality was notable. The 30-day mortality rate was 5% in mTBI compared to 2.8% in NTBI (*p* < 0.001), representing a threefold difference. This is considerable for a condition labelled 'mild,' particularly given that 76% of patients met criteria for complicated mTBI, with severe intracranial injuries frequently present. The definition of mTBI has evolved over time. It was originally limited to GCS 14–15 [16] but broadened to include GCS 13 following ATLS guidelines in 2008 [11], a change that remains debated [15, 26]. Our findings reinforce that GCS alone does not adequately reflect the extent of intracranial injury or predict short-term outcome.

Age remains the most established risk factor for adverse outcome after TBI, including in this cohort [17, 24, 25], but ASA class was independently associated with mortality even after controlling for age, confirming that pre-injury health exerts an effect beyond chronological age**.** The risk of death increased progressively with each higher ASA category, consistent with our previous findings [8, 9]. The importance of pre-injury health has received growing attention as TBI populations shift demographically in many parts of the world [3, 4] but its impact remains debated. Some studies report associations between pre-existing health conditions and in-hospital mortality [2], some report conflicting results regarding 90-day mortality [9, 18], and some studies identify correlations only with 1-year mortality [27]. Research on frailty and TBI outcomes has shown similar variability [20].

ASA class has previously been shown to be associated with mortality in trauma populations more broadly. In a large cohort of trauma patients, Skaga et al. demonstrated that higher ASA class was independently associated with increased mortality, even after adjustment for injury severity and physiological parameters [22]. Subsequent studies have reported similar findings, supporting ASA as a marker of baseline vulnerability rather than comorbidity alone [12]. The present study extends this body of work by demonstrating that this association is also evident in patients with mild traumatic brain injury, a group traditionally considered to have low short-term mortality risk, and by confirming the association at a national level.

A recognized limitation across these studies has been differences in how comorbidity and frailty are defined and measured. The use of ASA class, a widely applied proxy for both comorbidity and frailty [14, 19], may help address this limitation. ASA class was also significantly associated with mortality in the NTBI cohort, in contrast to our previous study [9]. As discussed in that study, the lack of significance was likely attributable to the smaller sample size, and the odds ratio suggested a trend toward association. With 26,001 NTBI patients in the current study, statistical power was sufficient to detect this effect. Although the direction and graded pattern of the association were similar, the relative contribution of injury-related variables differed between cohorts. In this study, we included GCS, mechanism of injury, and AIS head score or NISS in the multivariable analysis alongside age and ASA class. Despite this adjustment for injury severity and mechanism, ASA remained independently associated with mortality. This indicates that pre-injury health provides information beyond traditional trauma severity metrics, and that its effect persists when accounting for the anatomical severity of brain injury and level of consciousness.

Mechanism of injury, specifically low-energy falls (LEF), has been proposed as a marker for frailty in hip fracture populations [6], and LEF was associated with mortality in univariable but not multivariable analysis for mTBI. This suggests that in mTBI, mechanism may indeed function as a marker of underlying frailty and comorbidity burden rather than as an independent risk factor. Despite the similarity in ASA class’s' effect across injury types, LEF remained independently associated with mortality in the NTBI cohort. A possible explanation could be that LEF-associated injuries in NTBI patients (such as hip or pelvic fractures) often require surgery, adding operative stress and immobilization risk not fully captured by injury severity scores. In contrast, for mTBI patients, the relationship between brain injury and its complications appears to be sufficiently correlated with pre-injury health and age that the mechanism becomes less informative once these factors are accounted for.

A substantial proportion of the mTBI cohort had severe intracranial injuries, with 30% of the deceased group having an AIS head score of 5, compared to 4.6% of survivors. The AIS head score showed a dose–response relationship with mortality and remained independently associated with mortality in multivariable analysis. This indicates that anatomical injury severity contributes independently to outcome. The high proportion of complicated mTBI likely reflects both the registry inclusion criteria and the demographic shift toward elderly patients, in whom even low-energy mechanisms can produce severe intracranial injuries.

Polytrauma remained independently associated with mortality in the mTBI cohort after full adjustment. In contrast, isolated TBI was associated with mortality in crude analysis but was no longer independently associated after adjustment for age, pre-injury health, and intracranial injury severity. This pattern suggests that the crude association with isolated TBI is driven by differences in baseline vulnerability rather than the injury pattern itself. Polytrauma, on the other hand, adds physiological stress, can require surgery or immobilization, and increases the risk of complications such as infection or thromboembolism. The independent association between polytrauma and mortality suggests that even relatively minor extracranial injuries can worsen outcomes in vulnerable mTBI patients. Polytrauma was not significant in the NTBI cohort, which could reflect reduced observed mortality associated with polytrauma when patients with head injury are excluded. Extracranial polytrauma seems to have limited impact on mortality in otherwise mild and moderate trauma.

An important consideration is whether treatment bias may influence the observed mortality patterns. Research has documented that treatment intensity is often reduced in elderly patients compared to younger adults with similar injury severity [21], and under-triaging of elderly trauma patients is well documented [1, 10]. Given that many centers lack neurosurgical capabilities and the threshold for transferring an mTBI patient to a higher level of care may differ from other injuries, variations in care intensity could contribute to observed mortality patterns. Age-based assumptions about prognosis may influence decisions regarding care intensity or withdrawal of support in elderly mTBI patients. While our data cannot directly assess treatment intensity or decision-making patterns, this remains a consideration for interpretation and warrants investigation in future studies.

Several limitations should be acknowledged. First, ASA scoring has known inter-rater variability and involves subjective clinical judgment [14]. Different clinicians may assign different scores to the same patient, and the ASA class may be influenced by the clinical context. However, ASA class remains routinely collected in clinical practice and, given the formal criteria, appears to capture both frailty and comorbidities. The observation of consistent associations across studies despite this measurement variability suggests that the underlying signal is robust. Second, as with retrospective registry-based studies, there are inherent limitations related to data quality and completeness. The potential for selection bias exists, as well as the potential for incorrect documentation, missing data, or inconsistent coding practices across centers and over time. As multivariable analyses were based on complete-case data, the generalizability might be limited if missingness was not completely at random. However, SweTrau has been validated [5], which provides some assurance regarding data quality. Third, our 30-day mortality endpoint may not capture the full burden of mortality after mTBI. Our previous study showed that 55% of deaths occurred between 30- and 90-days post-injury, and the influence of pre-injury health is likely to increase with longer follow-up. The current findings may therefore represent a conservative estimate of ASA's prognostic value. Fourth, we lacked cause-of-death data to distinguish whether mortality was directly trauma-related, attributable to decompensation of pre-existing comorbidities, or due to in-hospital complications. Additionally, patients with high ASA class have elevated baseline mortality risk, and we cannot fully separate trauma-attributable deaths from expected mortality in this population. Finally, we could not assess treatment intensity or withdrawal of life support decisions, which may influence observed mortality and differ by age and ASA class. The potential for treatment bias warrants investigation in future studies.

## Conclusion

In this nationwide cohort of patients with mild traumatic brain injury, pre-injury health status measured by the ASA class was independently associated with 30-day mortality after adjustment for age and injury-related factors. Similar graded associations observed in non-TBI trauma suggest that comorbidity and baseline health contribute to early post-trauma survival across injury types. While ASA is not a measure of injury severity, its routine availability and consistent association with mortality indicate that it may provide complementary information alongside traditional clinical and anatomical assessments, particularly in older patients with multiple comorbidities. Further studies are needed to confirm these findings in other settings and to explore whether early identification of high-risk patients based on pre-injury health can meaningfully inform clinical decision-making.

## Supplementary Information

Below is the link to the electronic supplementary material.

ESM 1 Supplementary Material 1 (DOCX 80.3 KB)

## Data Availability

The data that support the findings of this study are available on request from the corresponding author OK. The data are not publicly available due to them containing information that could compromise research participant privacy.
