# Supplementary material for: ASA class is independently associated with 30-day mortality after mild traumatic brain injury with intracranial injury: a national retrospective cohort study
Source: Acta Neurochir (Wien). 2026 May 9;168(1):102. doi: 10.1007/s00701-026-06900-9 (PMC13157432; doi:10.1007/s00701-026-06900-9)
Supplement: Supplementary file 1 — Supplementary Material 1 (DOCX 80.3 KB) [file 701_2026_6900_MOESM1_ESM.pdf]

Supplementary table 1: ICD-10 codes defining TBI

S06.0	Concussion
S06.1	Traumatic cerebral oedema
S06.2	Diffuse brain injury
S06.3	Focal brain injury
S06.4	Epidural hemorrhage
S06.5	Traumatic subdural hemorrhage
S06.6	Traumatic subarachnoid hemorrhage
S06.7	Intracranial injury with prolonged coma
S06.8	Other intracranial injuries
S06.9	Intracranial injury, unspecified
